# Clinical and Functional Outcomes of Platelet-Rich Plasma Injection and Corticosteroid Injection in Patients With De Quervain’s Tenosynovitis: A Comparative Retrospective Study

**DOI:** 10.7759/cureus.66824

**Published:** 2024-08-13

**Authors:** Rohith C Sunil, Hariprasad Seenappa, Nagakumar J S, Siyad M Nazar, Tarun Kumar Somisetty

**Affiliations:** 1 Department of Orthopedics, Sri Devaraj Urs Academy of Higher Education and Research, Kolar, IND

**Keywords:** treatment efficacy, musculoskeletal disorders, functional outcomes, clinical outcomes, comparative study, corticosteroid injection, platelet-rich plasma (prp) injection, de quervain’s tenosynovitis

## Abstract

Background and aim

De Quervain’s tenosynovitis (DQST) is a prevalent condition involving tendon inflammation in the wrist. This study compares the clinical and functional outcomes between patients receiving platelet-rich plasma (PRP) injections and those receiving corticosteroid injections for DQST.

Methods

A retrospective study conducted at Sri Devaraj Urs Academy of Higher Education and Research, Kolar, India, included 58 patients with DQST, divided into Group A (PRP injection) and Group B (corticosteroid injection). Assessments were conducted using the Visual Analogue Scale (VAS), the Disabilities of the Arm, Shoulder, and Hand (DASH) score, and the Modified Mayo Wrist Score (MMWS) at baseline, one month, three months, and six months. Statistical analyses were performed using IBM SPSS Statistics for Windows, Version 22.0 (Released 2013; IBM Corp., Armonk, NY, USA), with significance set at p < 0.05.

Results

Both treatment groups demonstrated a reduction in VAS scores over time. Significant improvements were observed at one month (p = 0.007) and six months (p = 0.004) post-injection. Baseline (p = 0.336) and three-month (p = 0.829) VAS scores showed no significant differences. Similarly, DASH scores were not significantly different at any measured time points: baseline (p = 0.331), one month (p = 0.592), three months (p = 0.707), and six months (p = 0.314). MMWS scores also showed no significant differences at baseline (p = 0.123), one month (p = 0.101), three months (p = 0.422), and six months (p = 0.956). Independent sample t-tests highlighted significant VAS score improvements at one month (t = 2.813, p = 0.007) and six months (t = -3.009, p = 0.004), but DASH and MMWS scores showed no significant differences at any time points. Chi-square tests indicated no significant associations between the groups at one-month, three-month, and six-month follow-ups.

Conclusion

Both PRP and corticosteroid injections effectively alleviate pain in DQST patients, as evidenced by significant VAS score improvements. However, functional outcomes measured by DASH and MMWS scores did not significantly differ between the treatments. These results suggest that while both treatments are effective for pain management, their short-term impact on functional improvement may be similar. To investigate long-term functional results, more research with bigger sample sizes and longer follow-up periods is required.

## Introduction

Inflammation of the wrist tendons at the base of the thumb is known as de Quervain’s tenosynovitis (DQST), and it primarily affects the abductor pollicis longus and extensor pollicis brevis in the first extensor compartment [1]. It is often linked to repetitive or forceful manual tasks and pregnancy [2]. According to epidemiological data, the prevalence of DQST peaks in the 40s and 50s, affecting approximately 0.5% of men and 1.3% of women [3]. Patients usually report pain over the radial styloid process due to tendon friction in the narrowed compartment [4]. Clinically, tenderness is noted on the radial side of the wrist, with symptoms exacerbated by the Finkelstein test [5]. Nonoperative treatments [6], such as thumb spica splinting, non-steroidal anti-inflammatory drugs, therapeutic exercises, and corticosteroid injection [7], are primary management strategies [8,9]. Given DQST’s impact on work and daily activities, it is crucial to evaluate the efficacy of different treatments for optimal outcomes [10-13]. This study compares the clinical and functional outcomes of platelet-rich plasma (PRP) versus corticosteroid injections in DQST patients [4,12,14], aiming to highlight PRP’s potential benefits [15,16]. Through retrospective analysis, this research seeks to provide insights to guide clinicians in making evidence-based decisions and improve patient care.

## Materials and methods

This retrospective study, conducted at Sri Devaraj Urs Academy of Higher Education and Research, Kolar, India, analyzed data from patients treated with injections for DQST with at least six months of follow-up. Data were sourced from medical records. Patients were divided into two groups: Group A received PRP injections, and Group B received corticosteroid injections. Assessments included the Finkelstein test, Visual Analogue Scale (VAS) [15], Disabilities of the Arm, Shoulder, and Hand (DASH) score, and Modified Mayo Wrist Score (MMWS) at baseline, one month, three months, and six months post-procedure in the outpatient department. The study included 58 patients meeting the criteria: aged 18 or older and treated for DQST between 2019 and 2024. Exclusion criteria were refusal to participate, allergies to injected products, psychiatric disorders affecting protocol compliance, local or systemic infections, coagulation disorders, myxedema, rheumatoid arthritis, gout, uncontrolled diabetes, history of steroid injection, initial carpometacarpal joint arthritis, Dupuytren’s contracture, prior wrist trauma, and pregnancy.

Statistical analysis

A Microsoft Excel spreadsheet was used to enter the data (Microsoft Corporation, Redmond, WA, USA), and IBM SPSS Statistics for Windows, Version 22.0 (Released 2013; IBM Corp., Armonk, NY, USA) was used for analysis. The chi-square test was used to determine the significance of the frequency and proportional presentations of the categorical data. The mean and standard deviation of continuous data were presented, and the mean differences between groups were ascertained using independent t-tests. Less than 0.05 was the threshold for statistical significance.

## Results

A total of 58 participants were enrolled in the study, with each group consisting of 29 individuals. In the PRP injection group, 72.4% of the cases involved the right side, with a male proportion of 51.7% and a female proportion of 48.3%. In the corticosteroid injection group, 69.0% of the cases involved the right side, with 65.5% being males and 34.5% being females. The average age of participants was 43.3 years, with a standard deviation of ±8.6.

Table 1 displays the VAS, DASH, and MMWS scores for the group that received corticosteroid injections, measured at baseline, one month, three months, and six months after treatment. The initial VAS scores had an average of 6.69, which was reduced to 1.66 by the six-month mark. Similarly, the average DASH score dropped from 28.86 at baseline to 4.17 at six months. The MMWS scores improved significantly from a baseline mean of 64.86 to 87.38 at six months. These findings demonstrate substantial improvements in pain (VAS), disability (DASH), and wrist function (MMWS) over six months, highlighting the effectiveness of corticosteroid treatment in improving patient outcomes.

**Table 1:** Comparison of VAS, DASH score, and MMWS among the group that received corticosteroids DASH, Disabilities of the Arm, Shoulder, and Hand; MMWS, Modified Mayo Wrist Score; VAS, Visual Analogue Scale

Scores	Minimum	Maximum	Mean	Standard deviation
VAS baseline	5	8	6.69	6.69
VAS one month	3	4	3.34	3.34
VAS three months	2	4	2.48	2.48
VAS six months	1	4	1.66	1.66
DASH baseline	20	38	28.86	26.86
DASH one month	9	21	13.34	13.34
DASH three months	4	15	7.93	7.93
DASH six months	2	8	4.17	4.17
MMWS baseline	54	77	64.86	64.86
MMWS one month	64	86	74.41	74.41
MMWS three months	73	89	81.45	81.45
MMWS six months	79	98	87.38	87.38

Table 2 presents data on VAS, DASH, and MMWS scores for patients treated with PRP at baseline, one month, three months, and six months post-treatment. The average baseline VAS score was 6.9, which was reduced to 1.14 by the six-month mark. The DASH scores started at an average of 27.93 and dropped to 3.66 after six months. The MMWS scores improved from a baseline mean of 62.62 to 87.45 at six months. These findings indicate significant improvements in pain (VAS), disability (DASH), and wrist function (MMWS) over six months, demonstrating the effectiveness of PRP treatment in improving patient outcomes.

**Table 2:** Comparison of VAS, DASH score, and MMWS among the group that received PRP DASH, Disabilities of the Arm, Shoulder, and Hand; MMWS, Modified Mayo Wrist Score; PRP, platelet-rich plasma; VAS, Visual Analogue Scale

Scores	Minimum	Maximum	Mean	Standard deviation
VAS baseline	5	8	6.9	0.772
VAS one month	2	6	3.9	0.939
VAS three months	1	4	2.52	0.634
VAS six months	0	2	1.14	0.441
DASH baseline	20	36	27.93	3.184
DASH one month	8	22	13.86	4.077
DASH three months	4	16	8.24	3.28
DASH six months	2	9	3.66	1.857
MMWS baseline	53	72	62.62	4.555
MMWS one month	6	86	69.66	13.805
MMWS three months	6	92	79.1	14.766
MMWS six months	81	96	87.45	4.005

Descriptive statistics for the VAS, DASH, and MMWS at baseline, one month, three months, and six months for both groups revealed a decrease in VAS scores over time, with significant improvements at one month (p = 0.007) and six months (p = 0.004). No significant differences were found in VAS scores at baseline (p = 0.336) and three months (p = 0.829). DASH scores did not differ significantly at any time points: baseline (p = 0.331), one month (p = 0.592), three months (p = 0.707), and six months (p = 0.314). Similarly, MMWS scores showed no significant differences at baseline (p = 0.123), one month (p = 0.101), three months (p = 0.422), and six months (p = 0.956).

Independent sample t-tests were used to compare the outcomes between the PRP and steroid groups, as shown in Table 3. Significant improvements in VAS scores were noted at one month (t = 2.813, p = 0.007) and six months (t = -3.009, p = 0.004). However, DASH and MMWS scores did not show significant differences at any time points, indicating that while pain reduction was achieved, functional improvements were not significantly different between the groups.

**Table 3:** Statistical analysis of VAS, DASH, and MMWS scores pre- and post-intervention DASH, Disabilities of the Arm, Shoulder, and Hand; MMWS, Modified Mayo Wrist Score; VAS, Visual Analogue Scale

Measure	T	df	Sig (two-tailed)	Mean difference	95% confidence interval
VAS baseline	0.971	56	0.336	0.207	-0.220 to 0.634
VAS one month	2.813	41.883	0.007	0.552	0.156 to 0.948
VAS three months	0.217	56	0.829	0.034	-0.284 to 0.353
VAS six months	-3.009	43.14	0.004	-0.517	-0.864 to -0.171
DASH baseline	0.98	56	0.331	1.069	-1.115 to 3.253
DASH one month	0.539	56	0.592	0.517	-1.405 to 2.440
DASH three months	0.378	56	0.707	0.31	-1.334 to 1.955
DASH six months	-1.015	56	0.314	-0.517	-1.538 to 0.503
MMWS baseline	-1.565	56	0.123	-2.241	-5.111 to 0.628
MMWS one month	-1.667	56	0.101	-4.759	-10.477 to 0.960
MMWS three months	-0.809	56	0.422	-2.345	-8.152 to 3.462
MMWS six months	0.055	56	0.956	-0.069	-2.424 to 2.562

## Discussion

The objective of the study was to compare the clinical and functional outcomes of PRP injections [17] versus corticosteroid injections in patients with DQST [18,19]. The results indicate that both treatments are effective in reducing pain, as shown by significant improvements in VAS scores at one month and six months post-treatment [20]. However, no significant differences were observed in functional outcomes as assessed by DASH and MMWS scores.

Pain reduction

Chi-square tests for the one-month, three-month, and six-month follow-ups revealed no significant associations, indicating no significant differences in these categorical outcomes between the treatment groups.

The notable reduction in VAS scores at one month (t = 2.813, p = 0.007) and six months (t = -3.009, p = 0.004) post-treatment underscores the effectiveness of both PRP and corticosteroid injections in reducing pain associated with DQST. This result is consistent with earlier research showing corticosteroids as an effective short-term solution for pain relief in DQST. For example, Lane et al. found that corticosteroid injections significantly alleviated pain within the first month of treatment, which supports our study’s findings [3]. Similarly, Ilyas observed significant pain reduction at six weeks post-treatment with corticosteroid injections, further affirming the short-term efficacy of corticosteroids [21].

Functional outcomes

Although significant pain relief was achieved, there were no notable differences in DASH and MMWS scores at baseline, one month, three months, and six months. This suggests that while both treatments are effective for pain management, they might not lead to substantial short-term improvements in functional outcomes. This observation is supported by a study by Peters-Veluthamaningal et al., which found that corticosteroid injections significantly reduced pain in DQST patients but did not significantly enhance functional outcomes compared to splinting alone [4]. Additionally, Fitzpatrick et al.’s study indicated that corticosteroid injections alleviated pain but had a limited effect on short-term functional recovery [22].

The absence of significant differences in DASH and MMWS scores implies that while PRP may relieve pain, it might not offer additional functional benefits over corticosteroid injections in the short term. This finding is crucial for clinical decision-making, highlighting the need to consider patient-specific factors and preferences when selecting between PRP and corticosteroid injections for managing DQST.

Limitations of the study

This study did not include a placebo-controlled group for comparison with the PRP plus steroid group. The findings may not be generalizable to the wider community due to the limited research site and small sample size. Additionally, there is very little literature available for comparison.

## Conclusions

The study concludes that both PRP and corticosteroid injections are effective for managing DQST, providing significant pain reduction and improved functional outcomes. However, corticosteroid injections were found to be slightly more effective for acute pain management (less than one month), while PRP showed slight improvement in the long term (six months). Overall, DQST was effectively managed, with no discernible difference between the two treatment regimens. The study highlights the need for more research to examine the long-term effects of PRP and corticosteroid injections on functional results, but it also supports its use for pain management in DQST. Larger sample numbers and longer follow-up times are required for future research in order to validate these results and get a deeper understanding of the mechanisms behind the treatment-dependent differences.
